# Ticks and associated pathogens collected from cats in Sicily and Calabria (Italy)

**DOI:** 10.1186/s13071-015-1128-3

**Published:** 2015-10-07

**Authors:** Maria-Grazia Pennisi, Maria-Flaminia Persichetti, Lorena Serrano, Laura Altet, Stefano Reale, Laura Gulotta, Laia Solano-Gallego

**Affiliations:** Dipartimento di Scienze Veterinarie, Università di Messina, Polo Universitario Annunziata, Messina, 98168 Italy; Dottorato di Ricerca Scienze Mediche Veterinarie, Università di Messina, Polo Universitario Annunziata, Messina, 98168 Italy; Vetgenomics, Edifici Eureka, PRUAB, Bellaterra 08193 Barcelona, Spain; Istituto Zooprofilattico Sperimentale della Sicilia, A. Mirri, Via G. Marinuzzi 3, Palermo, 90129 Italy; Veterinary practitioner, Via F. Crispi 56, Lipari 98055 Messina, Italy; Departament de Medicina i Cirurgia Animals. Facultat de Veterinaria, Universitat Autonoma de Barcelona. Bellaterra, Cerdanyola 08193 Barcelona, Spain

**Keywords:** Tick, Cat, PCR, *Ehrlichia*, *Rickettsia*, *Bartonella*, *Leishmania* and *Babesia*

## Abstract

**Background:**

Limited information is available about the species of ticks infesting the cat and the pathogens that they harbor. The aims of the present study were to identify the species of ticks removed from cats living in Sicily and Calabria (Italy) and to detect DNA of vector-borne pathogens in the same ticks.

**Findings:**

Morphological identification of 132 adult ticks collected throughout the year from cats was carried out. Real-time PCRs for *Hepatozoon felis*, Piroplasmid, *Ehrlichia/Anaplasma* spp., *Rickettsia* spp., *Bartonella* spp., *Mycoplasma* spp. and *Leishmania infantum* were performed from each individual tick.

Ticks belonging to *Rhipicephalus* (*R. sanguineus* sensu lato, *R. pusillus*) and *Ixodes* (*I. ricinus*, *I. ventalloi*) genera were identified. *Ixodes ventalloi* was the most frequently found tick species (47 %).

The positivity rate to at least one pathogen was 14.4 % (19/132 ticks). *Leishmania infantum*, *Rickettsia* spp. (*R. monacensis* and *R. helvetica*), *Bartonella* spp. (*B. clarridgeiae*), Piroplasmid (*Babesia vogeli*), and *Ehrlichia/Anaplasma* spp. (*E. canis*) DNAs were amplified in 8.3, 5.3, 1.5, 0.75 and 0.75 % of ticks, respectively. *Hepatozoon felis*, *Anaplasma* spp. and hemotropic *Mycoplasma* spp. DNAs were not detected. Four (21.1 %) out of nineteen positive ticks were co-infected.

**Conclusions:**

This study provides novel data about ticks infesting cats and the DNA of pathogens that they harbor. In Southern Italy, anti-tick prophylaxis should be implemented throughout the year in cats without neglecting winter time.

## Findings

Ticks (Acari: Ixodida) are vectors of many pathogens (VBPs) some of them considered emerging and worldwide spread [1]. Moreover, a zoonotic concern is associated with some agents such as *Bartonella* spp.*, Rickettsia* spp.*, Ehrlichia* spp.*, Babesia* spp. and *Anaplasma phagocytophilum* [2]. In South Italy, the climate favors different tick species as previously described by some authors [3, 4]. However, limited information is available about the species of ticks infesting the cat and the pathogens that they harbor [1].

The aims of the present study were to identify the tick species removed from cats living in South Italy and to detect the DNA of some vector-borne pathogens in the same arthropods.

### Methods

One hundred and thirty two ticks were collected between November 2011 and March 2013 throughout the year in three sites: Lipari (Eolian archipelago, Sicily, 38° 28’ 3’ N, 14° 57’ 14’ E), Reggio Calabria (Calabria, 38° 06’ N, 15° 39’ E) and Gioia Tauro (Calabria, (38°25’30’ N, 15° 53’ 51’ E). Ticks were removed by a veterinarian as a preventative measure from outdoor owned cats during consultation in Lipari (*n* = 60), in Reggio Calabria (*n* = 20) and in Gioia Tauro (2) and from stray cats included in trap-neuter-release programs in Lipari (*n* = 130), Reggio Calabria (*n* = 77) and Gioia Tauro (*n* = 19) during the physical examination. Therefore, ethical committee approval was not needed. Informed consent was obtained from all owners and from the legal representative of animal welfare groups in charge of the management of stray cats.

Collected ticks were stored up in alcohol 70°. Date of collection and place of residence of the cat were recorded. Tick species and instars were determined on the basis of morphometric characteristics following conventional keys and descriptions [5–7]. Tick gender and feeding status in adult ticks (engorged/not engorged) were also evaluated. DNA extraction was performed using High Pure PCR Template preparation kit (Roche, Mannheim, Germany) according to the manufacturer’s instructions with some modifications [8]. *Leishmania infantum* real-time PCR test targeted the constant region in the minicircle kinetoplast DNA (NCBI accession number AF291093) [9]. A quantitative real-time PCR was performed as described [10]. Real-time PCR targeting *Hepatozoon felis,* hemotropic *Mycoplasma* spp., *Ehrlichia/Anaplasma* spp., Piroplasmid, *Rickettsia* spp. and *Bartonella* spp. was performed as described previously [11–14]. The target amplified for each pathogen and the used primers as well as details regarding tick genomic DNA amplification are shown in Table 1. Each positive product of the real-time PCR was sequenced by the BigDye Terminator Cycle Sequencing Ready reaction Kit (AB, Life Technologies) using the same primers. Sequences obtained were compared with GenBank database (www.ncbi.nlm.nih.gov/BLAST). Statistical differences (*P* value <0.05) between positivity to at least one real-time PCR and tick genus, engorgement and gender were analysed by the chi-square or Fisher’s exact test using GraphPad Instat software. Associations were evaluated using Odds Ratio (OR).

**Table 1:** Primers used for pathogen detection and tick genomic DNA amplification^a^

Pathogen	Region amplified	Primer Forward (5’-3’)	Primer Reverse (5’-3’)	Final [primer] (μM)	PCR Product (bp)	Reference
*Hepatozoon felis*	18S rRNA	CTTACCGTGGCAGTGACGGT	TGTTATTTCTTGTCACTACCTCTCTTATGC	0.3	146	[11]
*Ehrlichia/ Anaplasma* spp.	16S rRNA	GCAAGCYTAACACATGCAAGTCG	CTACTAGGTAGATTCCTAYGCATTACTCACC	0.5	102^b^	[11]
Piroplasmid	18S rRNA	GACGATCAGATACCGTCGTAGTCC	CAGAACCCAAAGACTTTGATTTCTCTC	0.3	114^b^	VetGenomic In-house design
*Rickettsia* spp.	ITS1	GCTCGATTGRTTTACTTTGCTGTGAG	CATGCTATAACCACCAAGCTAGCAATAC	0.5/0.3	300^b^	[11]
*Bartonella* spp.	ITS1	AGATGATGATCCCAAGCCTTCTG	CCTCCGACCTCACGCTTATCA	0.3	180^b^	Modified from [12] and [13]
Hemotropic *Mycoplasma* spp.	16S	GGAATCACTAGTAATCCYGTGTCAGCTATAT	GGCGGTGTGTACAAGCCTGG	0.3	187^b^	[14]

^a^The eukaryotic 18S RNA Pre-Developed TaqMan Assay Reagents (AB, Life technologies) was used as an internal reference for genomic DNA amplification to ensure the proper PCR amplification of each sample. ^b^Targeted size could vary depending on the species

## Results

Results of tick species identification, season of collection, number, gender and feeding status in female ticks are listed in Table 2.

**Table 2:** Tick species identified, season of collection, number, gender and feeding status of ticks

Tick species and season of collection	Number of male ticks (%)	Number of female ticks (%) [% of ticks engorged]	Total
*Ixodes ventalloi* ^a,b,d^	12 (19)	50 (81) [88]	62
*Ixodes ricinus* ^a,b,d^	13 (65)	7 (35) [100]	20
*Ixodes* spp.^a,b,d^	0 (0)	5 (100) [100]	5
*Rhipicephalus sanguineus* sensu lato^b,c^	14 (50)	14 (50) [42,8]	28
*Rhipicephalus pusillus* ^b,c^	17 (100)	0 (0) [0]	17
TOTAL	56 (42)	76 (58) [81,5]	132

^a^Winter; ^b^Spring; ^c^Summer; ^d^Autumn. No male tick was engorged

Majority of ticks (*n* = 128) were removed from 18 % (35/190) of cats evaluated at Lipari, with a range of 1–22 ticks/cat. In Calabria, ticks were collected from 2 out of 118 cats only (1.7 %). Three *R. sanguineus* s.l. tick specimens were removed from a cat in Reggio Calabria and one *I. ricinus* tick from a cat living in Gioia Tauro.

Different tick species of the same genus were frequently collected from a single cat. Three cats, sampled between March and May, were infested by both *Rhipicephalus* and *Ixodes* ticks. One of these latter cats was found infested by all four tick species detected in this study.

The tick positivity rate to at least one pathogen (*Bartonella* spp., *Rickettsia* spp., *Ehrlichia/Anaplasma* spp., Piroplasmid and *L. infantum*) was 14.4 % (19/132 ticks) (Table 3). This positivity rate was respectively 8.9 % in male (5/56) and 18.4 % in female (14/76) ticks. Moreover, 2 out of 13 non-engorged female ticks (15.3 %) and 10 out of 63 engorged female ticks (15.8 %) were positive to at least one pathogen.

**Table 3:** Pathogen PCR results and GenBank ID sequences according to tick species

	Number of positive ticks to any pathogen/ numbers of ticks studied (%)						
PCR pathogens	*I. ventalloi*	*R. sanguineus* sensu lato	*R. pusillus*	*I. ricinus*	*Ixodes* spp.	Ticks total (%)	GenBank ID sequences
*Bartonella clarridgeiae*	1/62 (1.6 %)	1/28 (3.5 %)	0 (0 %)	0 (0 %)	0 (0 %)	2/132 (1.5 %)	emb|FN645454.1|
Hemotropic *Mycoplasma* spp.	0 (0 %)	0 (0 %)	0 (0 %)	0 (0 %)	0 (0 %)	0/132 (0 %)	NA
*Rickettsia* spp.	1/62 (1.6 %)^a^	2/28 (7.1 %)	0 (0 %)	2/20 (10 %)	2/5 (40 %)	7/132 (5.3 %)	ND
*Rickettsia monacensis*	1 (1.6 %)	2 (7.1 %)	0 (0 %)	1 (5 %)	1 (20 %)	5 (3.8 %)	gb|KF016136.1|
Rickettsia helvetica	1 (1.6 %)	0 (0 %)	0 (0 %)	1 (5 %)	1 (20 %)	3 (2.3 %)	gb|JQ796866.1|
*Ehrlichia* spp. /*Anaplasma* spp.	1/62 (1.6 %)	0 (0 %)	0 (0 %)	0 (0 %)	0 (0 %)	1/132 (0.75 %)	*Ehrlichia canis* KF034789.1
*Babesia vogeli*	0 (0 %)	1/28 (3.5 %)	0 (0 %)	0 (0 %)	0 (0 %)	1/132 (0.75 %)	JX871885.1
*Hepatozoon felis*	0 (0 %)	0 (0 %)	0 (0 %)	0 (0 %)	0 (0 %)	0/132	ND
*Leishmania infantum*	4/62 (6.4 %)^b^	3/28 (10.7 %)^c^	3/17 (17.6 %)	1/20 (10 %)	0 (0 %)	11/132 (8.3 %)	ND
Total number of positive ticks to any pathogen/total numbers of ticks studied (%)	6/62 (9.7 %)	5/28 (17.8 %)	3/17 (17.6 %)	3/20 (15.0 %)	2/5 (40.0 %)	19/132 (14.4 %)	NA

^a^One tick was co-infected with *R. monacensis* and *R. helvetica*; ^b^one female tick co-infected with *B. clarridgeiae*. ^c^One male tick coinfected with *B. vogeli* and one female tick co-infected with *R. monacensis*. NA not applicable, ND not determined

No significant differences were found between positivity at least to one pathogen and tick genus, engorgement of females and gender.

*Leishmania infantum* DNA was found in 11 ticks (seven females and four males) and the median *Leishmania* parasite load was 200 parasites/specimen (range 17–555 *Leishmania*/specimen).

## Discussion

In the present study, active adult ticks were found on cats during all seasons in all sites of collection. However, almost all ticks were found on cats from Lipari (Eolian Archipelago). Interestingly, *I. ventalloi*, *I. ricinus*, *R. sanguineus* s.l. and *R. pusillus* ticks were collected from cats. There are no published data on ticks removed from cats in Sicily and Calabria but *Rhipicephalus* spp. ticks were the only tick species removed from dogs and represented the most prevalent ticks in Sicily [15]. Ecological factors, season of tick sampling, climatic variations and host preferences may be responsible for the differences observed. In Northwestern Italy, *R. sanguineus* s.l. was found in 86.5 % of infested dogs and 26.3 % of infested cats while *I. ricinus* infested 18.5 % of dogs and 68.4 % of cats [16]. *Ixodes ricinus* ticks were the most common ticks found on cats in Europe as north as the Artic Circle and this is one of the southernmost finding of these ticks in Europe [17–20]. Migrating birds are considered dispersal agents of larval stages of *I. ricinus* and they could contribute to the presence of this tick species in the studied areas which are stop-over and nesting sites of migratory birds moving from Africa to Central-Northern Europe [21].

*Ixodes ventalloi* ticks were the most prevalent tick species removed from cats in Lipari (48 % of tick specimens collected in this site) and for the first time it was found PCR positive to *L. infantum*. The so-called “rabbit tick” is scarcely reported on dogs and cats and it is usually found on wild mammals (rabbit, fox, hedgehog, etc.) as well as on birds [19, 22]. We think that the presence of *I. ventalloi* in cats from Lipari is due to the abundance of wild rabbits (*Oryctolagus cuniculus*) in the island (*Piano Faunistico-Venatorio della Regione Siciliana*, 2013–2018).

This faunal peculiarity in Lipari Island can also explain our finding of *R. pusillus* which is also typical of wild rabbits but it may be found in dogs and other domestic or wild mammals [22–24]. This is the first *bona fide* report of *R. pusillus* from cats in Italy where this tick has been identified in rabbits and in a hedgehog [23, 24]. Outdoor cats, as predators of bunnies or birds, may particularly be exposed to infestation from ectoparasites of their pray and act as a link between wildlife and synanthropic habitats. Recently, *R. pusillus* was also removed from human patients in Italy [25] and this tick species was found positive to Mediterranean spotted fever (MSF) group Rickettsiae [24]. Interestingly, in the present study, *R. pusillus* was found for the first time PCR positive to *L. infantum*.

In the present study, we added new data about the vectorial potential of *I. ventalloi* as we found a tick DNA positive for both *R. helvetica* and *R. monacensis* and some other positive for *B. clarridgeiae*, *E. canis* and *L. infantum.* Moreover, we detected for the first time *R. monacensis, B. clarridgeiae* and *B. vogeli* DNA in *R. sanguineus* s.l. ticks removed from cats.

Finally, we confirmed that co-infections are quite common in ticks and may be responsible for polimicrobial infections in susceptible hosts [19, 25]. In fact, we observed the presence of DNA of different pathogens (*B. vogeli, R. monacensis, B. clarridgeiae*) in three ticks (*I. ventalloi* and *R. sanguineus *s.l.) positive to *L. infantum* which was the most prevalent pathogen DNA found in this study while one *I. ventalloi* harbored both *R. monacensis* and *R. helvetica*.

In conclusion, the present study provides new data on ticks collected from cats and associated pathogens. Effective preventative measures against tick infestations should be strongly recommended to pet cat owners all year around in the South of Italy.

## Abbreviations

Bp: Base pairs
DNA: Deoxyribonucleic acid
MSF: Mediterranean spotted fever
OR: Odds ratio
PCR: Polymerase chain reaction
VBPs: Vector borne pathogens
